# Lighting Spectrum, Intensity, and Photoperiod Induce Distinct Photoresponses in *Chrysanthemum coronarium* Greens, Cultivated in CEA

**DOI:** 10.3390/plants15091394

**Published:** 2026-05-01

**Authors:** Akvilė Viršilė, Kristina Laužikė, Ieva Karpavičienė, Audrius Pukalskas, Giedrė Samuolienė

**Affiliations:** Lithuanian Research Centre for Agriculture and Forestry, 58344 Kėdainiai, Lithuania; kristina.lauzike@lammc.lt (K.L.); ieva.karpaviciene@lammc.lt (I.K.); audrius.pukalskas@lammc.lt (A.P.); giedre.samuoliene@lammc.lt (G.S.)

**Keywords:** antioxidant properties, chlorophylls, LED lighting parameters, light use efficiency, mineral elements, shungiku greens, underutilized leafy vegetables

## Abstract

In controlled-environment agriculture (CEA), light serves both as an energy source for photosynthesis and as a regulatory factor. However, the light responses of underutilized leafy greens are still not fully characterized compared with model crops such as lettuce. This study evaluated the effects of lighting parameters on the growth, metabolism, antioxidant properties, and mineral composition of *Chrysanthemum coronarium* (shungiku) greens cultivated hydroponically in CEA. Three parallel experiments were conducted, aiming to explore the effects of (I) light spectrum using red (R, 660 nm), blue (B, 447 nm), and combined RB light; (II) photoperiod, using 12, 16, and 24 h photoperiods at equal daily light integral; and 150, 200, 250, and 300 µmol m^−2^ s^−1^ photosynthetic photon flux density (PPFD) at 16 h photoperiod. RB light promoted the highest biomass accumulation and light use efficiency (LUE), while monochromatic red and blue light limited growth and reduced Fe and Zn contents. A 12 h photoperiod yielded the best results for leaf area, fresh weight, and LUE compared with 16 and 24 h photoperiods. Higher PPFD increased biomass, soluble sugars, antioxidant capacity, organic acids, and micronutrients, with peak LUE at 200 µmol m^−2^ s^−1^ instead of the maximum yield at 300 µmol m^−2^ s^−1^. These findings emphasize the importance of crop-specific and trait-oriented light optimization for underutilized leafy vegetables.

## 1. Introduction

Over the years, scientific literature has frequently described light as a “key”, “essential”, or “critical” factor influencing plant growth, morphology, photosynthesis, and metabolism [1,2,3,4]. In the past decade, the definition of light has evolved alongside advancements in controlled-environment agriculture and vertical farming technology. Authors frame light not only as a growth requirement but also as a multidimensional management tool to optimize various plant outcomes. Key conceptual frameworks include light as a limiting production factor [5,6], energy-intensive input [7,8], positioning light as both essential and economically challenging in CEA systems, and light as a control mechanism, tailoring yield and quality [9,10,11,12]. From the perspective of quantum physics, light is defined as electromagnetic radiation that exhibits wave–particle duality [13]. Although the interpretation of this dual nature of light has been a topic of philosophical debate in quantum mechanics for centuries and continues to be discussed today [14], horticultural lighting has successfully utilized this duality within an operational framework. Light wavelengths are used to characterize spectral composition [15], while photon-based metrics are employed to quantify light intensity and cumulative daily light integral [16]. This also reflects the multifaceted impacts of light on plant physiology. Light intensity, spectral composition, and photoperiod play crucial roles in regulating chlorophyll synthesis, tissue differentiation, cell wall composition, and sugar accumulation [3,4]. It is particularly important in controlled environment agriculture (CEA), where artificial light-emitting diode (LED) lighting serves as the sole source of light energy and information for plants. In CEA systems, artificial light is the primary energy input, making its management highly relevant to crop performance and resource use considerations. It also has high expectations for resource use efficiency and energy costs [17], requiring a multidimensional assessment of lighting parameters and their effects on plants.

Spectrum, photoperiod, and intensity are not interchangeable “lighting settings,” but rather distinct dimensions of plant regulation. There are a few publications that explore the impacts of all three dimensions of light in a single study on lettuce seedlings [18], spinach [19], and rocket [20]. All these studies indicate that the set of light parameters optimal for biomass yield is specific to the plant under investigation, and that there is a trade-off between the optimal light parameters for leafy vegetable growth and quality. Moreover, review papers on lettuce and other leafy greens indicate that no single lighting parameter is universally dominant in controlled-environment plant production, because responses are strongly species-, cultivar-, and goal-dependent [21,22,23]. Light intensity, typically measured as photosynthetic photon flux density (PPFD) and/or cumulative index, daily light integral (DLI), is the primary factor influencing productivity and biomass accumulation [22,24]. Spectral composition is more often identified as effective for steering morphology and quality-related traits, including protein content, sugars, antioxidant activity, and secondary metabolites such as β-carotene, vitamin C, anthocyanins, and tocopherols [21,23]. However, there is a divided consensus on whether full-spectrum white light or red–blue light in different ratios is more effective for CEA lettuce yield [25]. Photoperiod is generally interpreted as a practical tool for modulating daily light dose and energy use efficiency through its interaction with PPFD rather than as an isolated driver [22,24].

To optimize the productivity and nutritional quality of cultivated greens, a light recipe integrating all three lighting parameters should be developed in relation to the other cultivation conditions. Lettuce is widely regarded as a model crop for controlled-environment agriculture, including plant factories, and has therefore been extensively studied across diverse genotypes [26]. However, knowledge derived from lettuce cannot be directly extrapolated to other leafy green species. Leafy vegetables, due to their strong adaptability, short production cycle, and high market demand and thus high return on investment, are currently the primary crop for plant factories and vertical farms [26,27], aligning well with the economic and functional goals of CEA. However, their variable genotypes (from lettuce, spinach, arugula, and different *Brassicaceae* plants) [28] require species- and case-specific revisions to existing knowledge of plant lighting.

Moreover, an increasing number of species are gaining attention for cultivation in controlled-environment systems. Neglected and underutilized edible plants are increasingly viewed as important resources for diversifying modern horticulture, as they offer opportunities to improve dietary diversity and nutritional quality, thereby facilitating more resilient and sustainable horticultural and agro-food systems [29]. Among them, underutilized leafy vegetables are especially relevant because they often combine a rich nutritional and phytochemical profile with resilience to abiotic stress and relatively low crop input requirements [30,31]. Their introduction into controlled and soilless production systems could expand the crop portfolio of CEA while supporting higher water and nutrient use efficiency. These species are often rich in essential minerals, vitamins, and antioxidants and thus may help address micronutrient deficiencies associated with “hidden hunger”. It is stated that even diets rich in vegetables may not necessarily guarantee adequate intake of mineral micronutrients and phytonutrients, because nutritional adequacy depends not only on the amount of vegetables consumed, but also on their diversity and nutrient density, which may in some cases be diminished by dilution effects associated with high-yield production [32]. Nevertheless, the wider adoption of such crops depends on targeted species-specific research to define suitable agronomic practices, optimize productivity and quality, and address potential safety concerns.

*Chrysanthemum coronarium* L. (syn. *Glebionis coronaria*), or shungiku, is a promising candidate for CEA diversification. As an edible member of the *Asteraceae* family, it is related to lettuce yet remains much less studied in indoor systems. Reports on *G. coronaria* indicate that it contains a range of beneficial compounds, including phenolics, flavonoids, carotenoids, vitamin C, minerals, and various antioxidant-active phytochemicals such as sesquiterpene lactones and terpenes [33,34,35]. Additionally, other edible chrysanthemums have been found to contain specific flavonoids, including flavanomarein, flavanokanin, marein, and okanin, as well as two phenolic acids: chlorogenic acid and 3,5-dicaffeoylquinic acid [36]. The emerging tr of using underutilized vegetables extends beyond traditional cooking methods. These vegetables are now being investigated for their roles in functional foods, nutritional supplements, and even biofortified products [31]. *C. coronarium* has a long history of use as both a food plant and a source of bioactive plant materials. In Mediterranean countries, its extracts and essential oils are valued for their antioxidant, antimicrobial, insecticidal, and other functional properties. In East Asia, the plant is well known as an edible species with culinary and traditional value. The leaves are consumed as fresh or cooked greens, whereas the flowers are used in herbal infusions [35,37,38]. Evaluating *Chrysanthemum coronarium* in controlled environments could broaden CEA’s crop portfolio and determine whether cultivation techniques developed for lettuce can be applied to other edible greens within the same family. While this species is known from field and greenhouse cultivation, its responses to fully artificial lighting in hydroponic CEA remain poorly understood. To address this gap, the present study was designed as a comparative evaluation of three key lighting dimensions—spectrum, photoperiod, and intensity—through separate controlled experiments investigating their effects on growth, metabolism, and mineral element accumulation in *C. coronarium*.

## 2. Results

Lighting parameters had diverse effects on chrysanthemum growth (Table 1): plant height was the least affected, whereas leaf area and fresh and dry weight differed significantly between treatments. Monochromatic red (R) and blue (B) light reduced growth: leaf area was 50% and 22%, and dry plant weight was 41% and 17% lower than in the RB treatment, respectively. The photoperiod duration, while keeping the DLI (daily light integral) constant, had a pronounced impact only at the shortest investigated duration. Under a 12 h photoperiod, 53% larger leaf area and 46% and 35% higher fresh and dry plant weight, respectively, compared to the 16 h photoperiod were observed. A lighting intensity of 250 µmol m^−2^ s^−1^ was used as the reference, and a lower lighting intensity of 150 µmol m^−2^ s^−1^ resulted in 20% smaller leaf area and 20% and 27% lower fresh and dry weight, respectively. Higher lighting intensity (300 µmol m^−2^ s^−1^) was associated with greater growth: chrysanthemum greens had 27% larger leaf area, and 23% and 35% larger fresh and dry weight, respectively. Light use efficiency (LUE) differed significantly among lighting treatments, but the most efficient use of supplied light did not always coincide with the highest biomass production (Table 1). Combined RB light provided the highest LUE compared to monochromatic red and blue treatments, whereas monochromatic red light was the least efficient. At equal DLI, the shortest 12 h photoperiod with the highest light intensity resulted in significantly greater LUE than the 16 h and 24 h regimes. Under different light intensities, LUE was highest at 200 µmol m^−2^ s^−1^ and lowest at 250 m^−2^ s^−1^, while the 150 and 300 µmol m^−2^ s^−1^ treatments showed intermediate values.

**Figure 1:**
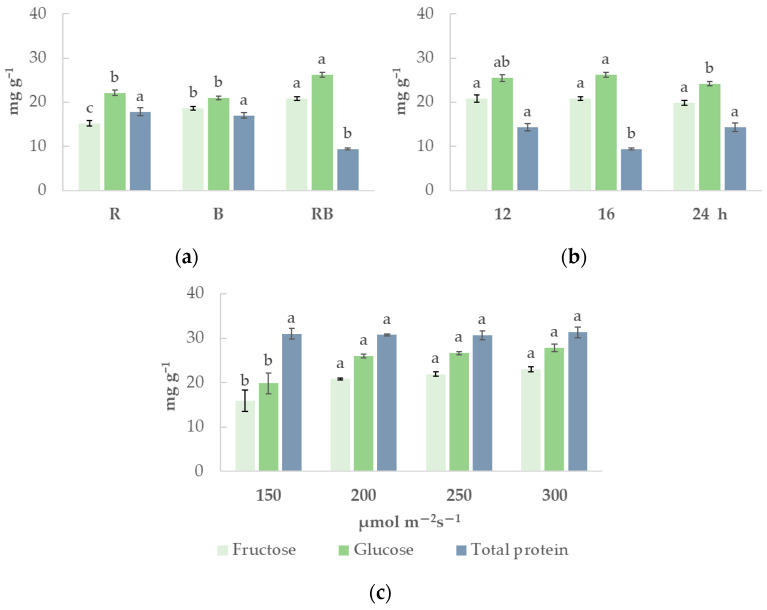
The content of sugars and proteins in *Chrysanthemum coronarium* under different lighting parameters ($\bar{x},\;n=3\;\mathrm{experimental}\;\mathrm{replications})$. Plants were cultivated under different lighting spectra (R—red light, B—blue light, RB—combination of red and blue light) (**a**), different photoperiods (12 h, 16 h, 24 h) (**b**) and under different light intensity (150, 200, 250, 300 μmol m^−2^ s^−1^) (**c**). Different lowercase letters indicate statistically significant differences between means of different lighting parameters in each experiment according to one-way ANOVA, Tukey’s test, when *p* ≤ 0.05.

The variation in primary metabolite contents (Figure 1) was also sensitive to the lighting conditions. Monochromatic red and blue lights resulted in 27% and 16% lower fructose and glucose levels, respectively, while total protein content was 89% and 81% higher than in the RB lighting treatment. Correlation analysis (Table S1A) showed strong positive (r from 0.80 to 0.91) correlation between fructose contents and growth parameters (leaf area, fresh and dry weight), as well as strong negative correlation (r from −0.80 to −0.90) between soluble sugar and total protein contents. These trends are not repeated across different photoperiod exposures (Table S1B). Lighting photoperiod and the same DLI had a pronounced impact only on protein content: it was 52% higher under short 12 h photoperiod and continuous 24 h lighting than under 16 h photoperiod. Light intensity, in contrast, did not affect total protein content, whereas low light intensity (150 µmol m^−2^ s^−1^) resulted in 27% and 25% lower fructose and glucose contents, respectively, compared to higher lighting treatments. Correlation analysis, similarly to light spectrum exposure (Table S1C), reveals strong positive relations between soluble sugar content and growth parameters, but no correlation between sugar and protein content.

**Figure 2:**
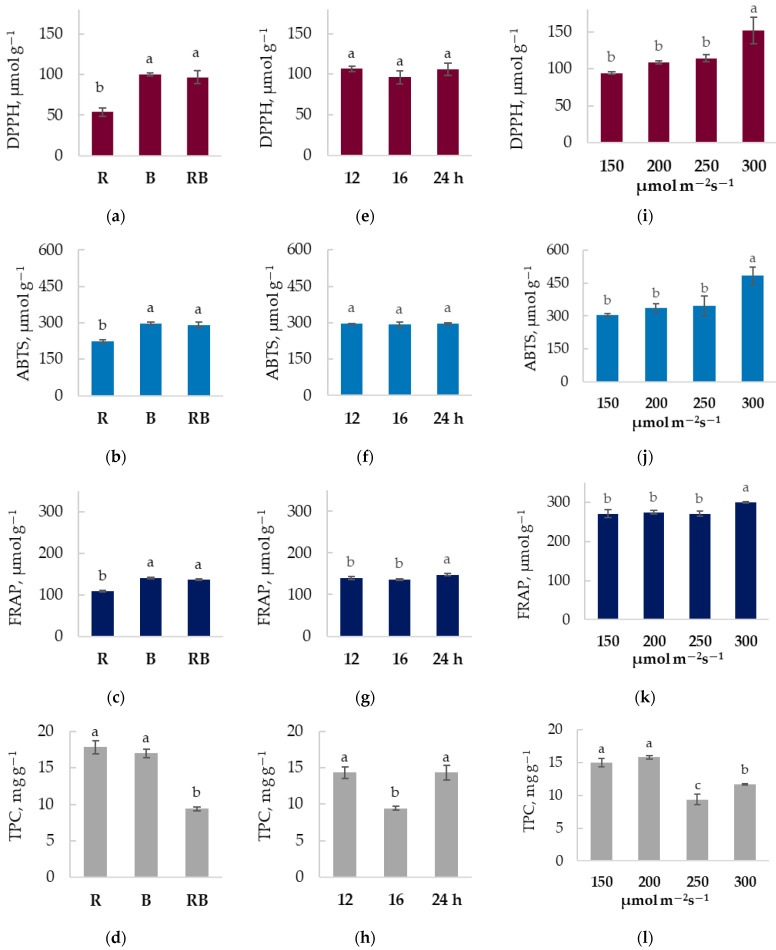
The lighting parameters impact on antioxidant activity indices in *Chrysanthemum coronarium* ($\bar{x}\pm SD,\;n=3\;\mathrm{biological}\;\mathrm{replications})$. *Chrysanthemum coronarium* were cultivated under different lighting spectra (R—red light, B—blue light, RB—combination of red and blue light) (**a**–**d**), different photoperiods (12 h, 16 h, 24 h) (**e**–**h**) and under different light intensity (150, 200, 250, 300 μmol m^−2^ s^−1^) (**i**–**l**) Different letters indicate statistically significant differences in the means of different lighting treatments according to one-way ANOVA, Tukey’s test, when *p* ≤ 0.05.

Antioxidant properties (Figure 2) also differ in chrysanthemum plants under contrasting lighting treatments. In the case of light spectra, monochromatic red light resulted in 44% and 23% lower DPPH and ABTS free radical scavenging activities, and 21% lower FRAP antioxidant power. Antioxidant properties in plants grown under blue light did not differ significantly from those in the RB treatment. At the same time, chrysanthemum leaves cultivated under monochromatic red and blue light contained 90% and 81% higher levels of total phenolic compounds, respectively. Correlation analysis (Table S1A) shows strong negative correlations between DPPH and ABTS free radical scavenging activities, FRAP, chlorophyll B, and malic and fumaric acid contents. Additionally, DPPH free radical activity negatively correlates with violaxanthin and lutein, while total phenolic content positively correlates with violaxanthin and β-carotene. These interrelations were not observed under photoperiodic exposure (Table S1B). In case of different photoperiod exposure, both shorter photoperiod (12 h) at higher PPFD and continuous lighting (24 h) at lower PPFD elevated total phenolic contents by 50% compared to 16 h photoperiod, but did not have a statistically significant impact on free radical scavenging activities and FRAP antioxidant power. The highest investigated lighting intensity of 300 µmol m^−2^ s^−1^ distinguished from lower light intensity treatments by 33, 40, and 11% higher DPPH, ABTS free radical scavenging activities, and FRAP antioxidant power. It also resulted in 25% higher total phenolic content compared to the 250 µmol m^−2^ s^−1^ PPFD treatment; however, chrysanthemum leaves under lower light intensities contained ~60% more phenolic compounds. Under different light intensities, DPPH and ABTS free radical scavenging activities correlate moderately, but negatively (r from −0.76 to −0.70) with chlorophyll B content (Table S1C).

Tocopherol, carotenoid, and chlorophyll contents (Table 2) varied under different lighting treatments. α-tocopherol varied significantly only with lighting photoperiod: it is higher at higher PPFDs during shorter photoperiods than under continuous 24 h lighting, and was ~50% higher at 12 h than at 24 h. Violaxanthin content was significantly higher only under monochromatic red (R) treatments (45% higher than in the RB treatment). At the same time, differences in lutein, β-carotene, and chlorophyll contents were more pronounced under photoperiod treatments. Higher PPFD at a shorter 12 h photoperiod exposure, as well as the highest investigated PPFD of 300 µmol m^−2^ s^−1^ in the lighting intensity experiment, was associated with 5−13% lower chlorophyll a and b contents.

**Figure 3:**
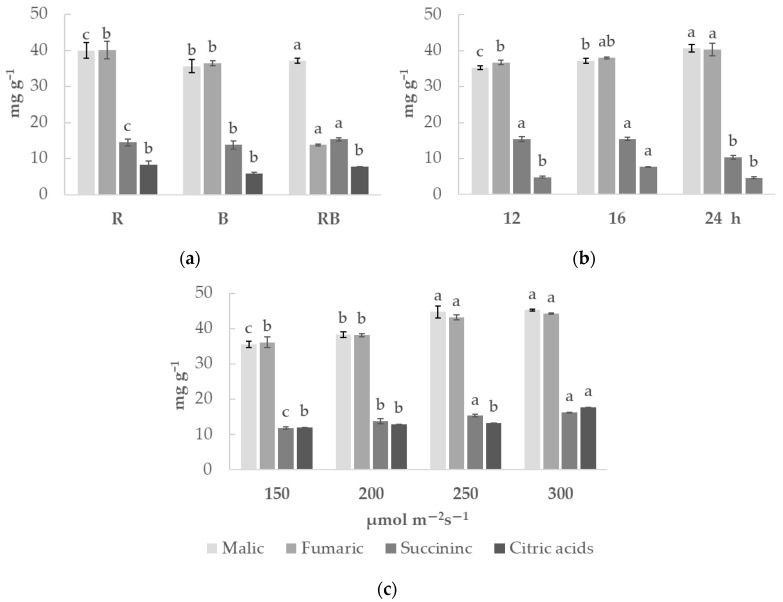
The lighting parameters impact on organic acid contents in *Chrysanthemum coronarium* ($\bar{x}\pm SD,\;n=3\;\mathrm{biological}\;\mathrm{replications})$. Plants were cultivated under different lighting spectra (R—red light, B—blue light, RB—combination of red and blue light) (**a**), different photoperiods (12 h, 16 h, 24 h) (**b**) and under different light intensity (150, 200, 250, 300 μmol m^−2^ s^−1^) (**c**). Different letters indicate statistically significant differences in the means of different lighting treatments according to one-way ANOVA, Tukey’s test, when *p* ≤ 0.05.

Organic acid contents (Figure 3) in chrysanthemum greens are also responsive to light. Under monochromatic red light treatment (R), malic and fumaric acid contents were slightly elevated, while in photoperiod exposure experiments, succinic acid was 34% lower under continuous 24 h photoperiod treatment, compared to 12–16 h. Under lighting intensity exposure, increasing lighting PPFD resulted in elevated levels of all four identified organic acids.

**Figure 4:**
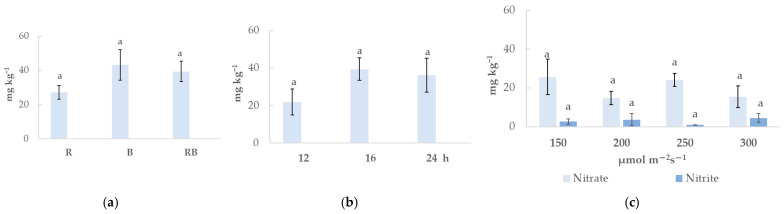
The lighting parameters impact on nitrate and nitrite contents in *Chrysanthemum coronarium* ($\bar{x}\pm SD,\;n=3\;\mathrm{experimental}\;\mathrm{replications})$. Plants were cultivated under different lighting spectra (R—red light, B—blue light, RB—combination of red and blue light) (**a**), different photoperiods (12 h, 16 h, 24 h) (**b**) and under different light intensity (150, 200, 250, 300 μmol m^−2^ s^−1^) (**c**). Different letters indicate statistically significant differences in the means of different lighting treatments according to one-way ANOVA, Tukey’s test, when *p* ≤ 0.05.

Though nitrate and nitrite contents (Figure 4) differ remarkably in chrysanthemum greens under different lighting treatments, due to high replication-to-replication variation, these differences were not statistically significant. Both red and blue monochromatic light were associated with significantly lower iron (34–70% lower content in leaves compared to RB lighting) and zinc (40–50% lower than under RB lighting) (Table 3). Monochromatic red additionally affected calcium (13% lower than RB) and blue affected manganese (17% lower than RB) contents. Under light spectra treatments, there is a moderate positive correlation (Table S1A) (r from 0.70 to 0.79) between Fe and fructose and glucose contents, while a strong negative correlation (r from −0.76 to −0.86) between Fe, Zn, and total protein, total phenolic compounds, and β−carotene contents. Correlation analysis also shows that Fe and Zn contents in chrysanthemum under different light spectrum treatments are strongly interrelated (r = 0.92). Plant leaf area, dry weight, and soluble sugar contents strongly and positively correlate with Zn contents. In contrast, Mn differently strongly negatively (r ~ −0.80) correlate with antioxidant properties and positively with chlorophyll b, malic, and fumaric acid contents.

Under photoperiodic lighting (Table 3), the response was also more pronounced for Fe and Zn content (2.3 and 1.3 times higher than the 16 h treatment) under continuous 24 h lighting. As in the spectra exposure, Fe is negatively correlated with sugar content (Table S1B). Still, a strong correlation was identified between Fe, chlorophylls, and organic acids, as well as between both Zn and Fe and other macroelement contents, which was not evident under light spectrum exposure.

The impact of lighting intensity (Table 3) was most pronounced on mineral element content. Lower light intensities of 150, 200 mol m^−2^ s^−1^ resulted in higher macroelement and lower microelement contents in chrysanthemum leaves, compared to higher lighting intensity. Similar to other lighting parameters, Fe, Zn, and, to a lesser extent, Mn contents were reduced under unfavorable lighting conditions. They were 27, 37, and 18% lower under 150 µmol m^−2^ s^−1^ lighting intensities than under 250 µmol m^−2^ s^−1^. All these elements are strongly and positively interrelated among themselves, as well as with growth parameters, soluble sugar content, and organic acid content (Table S1C). However, only Zn is correlated with the contents of other mineral elements. A moderate negative correlation was observed between Zn and K, Mg, and Na, whereas strong positive correlations were observed between Zn and Fe and Mn.

## 3. Discussion

Plant responses to environmental stimuli are strongly linked to their metabolic state, because acclimation and tolerance are mediated by stress-induced changes in primary and specialized metabolites and their associated signaling pathways [39,40,41]. Underutilized leafy vegetables show considerable diversity in metabolite composition compared to well-known leafy plants [42], suggesting that their nutritional and stress response traits should be evaluated on a crop- or genotype-specific basis rather than generalized across species. Therefore, developing plant-growing technologies for CEA systems is a complex challenge that requires a systematic approach, beginning with the definition of the optimal light spectrum [43].

### 3.1. Light Spectrum Effects

**Figure 5:**
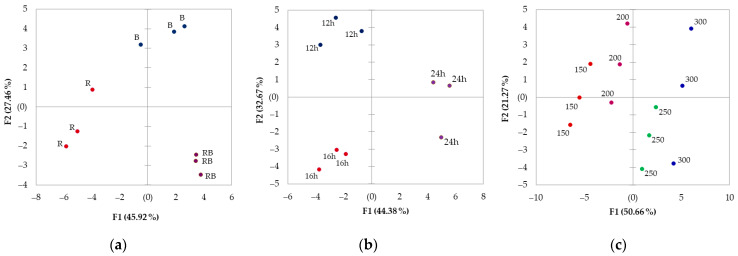
The Principal component analysis scatterplot of lighting parameters’ impact on *Chrysanthemum coronarium* (n=3 biological replications): lighting spectra (**a**), photoperiod (**b**), and intensity (**c**). Corresponding factor loadings are presented in Table S2.

The results of this study, summarized in principal component analysis (PCA) (Figure 5a), show that monochromatic red (according to F1) has a significantly different effect on chrysanthemum greens than monochromatic blue and combined RB. According to the factor loadings, these differences were mainly associated with growth parameters, soluble sugars, antioxidant response, chlorophyll a and b contents, malic and fumaric acids, and variation in Ca, Fe, and Zn (Table S2). According to F2, the red (R) and RB treatments differ from the blue (B) treatment in α tocopherol, lutein, β-carotene, succinic acid, citric acid, and Mn. The present results are broadly consistent with the trends reported in the research literature on leafy vegetable responses to lighting spectra. In lettuce and other leafy vegetables, such as spinach and kale, combining 75–83% of red light with 17–25% of blue has often provided a favorable balance between yield and resource use efficiency, while monochromatic light has been less effective for overall performance [43,44,45,46]. In the present study, *Chrysanthemum coronarium* responded most favorably to combined red–blue light, whereas monochromatic red and blue light reduced biomass formation, compared to RB, with monochromatic red having a more pronounced impact: 50% and 22% lower leaf area and 41% and 17% lower dry weight under R and B light, compared to the RB treatment. Mechanistically, blue light supports stomatal formation and opening, chlorophyll fluorescence, and photosynthetic regulation, whereas red light is highly efficient for photon capture but, when applied alone, may provide insufficient photoregulatory balance and induce morphological abnormalities; combined RB light therefore tends to support both carbon assimilation and coordinated development more effectively than either monochromatic treatment [44,47]. Other studies on leafy vegetables also confirm that monochromatic red and blue light produce distinctly different effects on lettuce and leafy vegetables across growth, metabolites, and mineral content [45,48]. In this study, monochromatic red resulted in lower fructose and glucose contents, 21–44% lower antioxidant activity, and significantly reduced Fe, Zn, Ca, and Mn contents. Although sole blue light was less inhibitory than sole red light, it still did not match RB for soluble sugar accumulation or mineral accumulation. Moreover, although monochromatic red and blue increased total phenolic content by 90–80%, relative to RB, this did not translate into consistently higher antioxidant capacity, especially under red light. This suggests that, in *Chrysanthemum coronarium*, as in lettuce, total phenolic concentration is not, by itself, a sufficient predictor of antioxidant performance; rather, the antioxidant response likely depends on the composition of phenolic compounds and on contributions from other antioxidant metabolites, pigments, and redox-active constituents. Within the limits of the present dataset, the stronger growth inhibition under monochromatic treatments also suggests that shungiku may be less tolerant than lettuce to spectral simplification [22,49]. Another study on underutilized leafy vegetable species, *Cichorium intybus* and *Cichorium endivia*, also concluded that genotype had a greater effect on overall physiological variation than did different R:B light ratios, suggesting a differential approach to regulating light parameters compared with traditional leafy crops [50].

Reported light spectrum effects on mineral element accumulation are less uniform than on growth responses. In spinach, the R:B light ratio modified Fe, Zn, Cu, and Mn contents, but these effects depended strongly on cultivar and Fe nutrition status, indicating that light-driven mineral responses are highly context-dependent [46]. In hydroponically cultivated lettuce, R:B ratio affected N, Ca, Mg, and K accumulation in leaves [51]. Another study also found that light spectral composition altered macro- and micronutrient uptake, including K, Ca, Mg, Fe, and Zn, and that the spectrum favoring mineral accumulation was not necessarily identical to that favoring biomass production [52]. Our results show that both Fe and Zn declined markedly (30–70%) under monochromatic red and blue compared with RB, and these decreases were positively associated with biomass and soluble sugars. This pattern may reflect coordinated responses of carbon metabolism and mineral acquisition to light quality rather than a direct causal relationship between Fe and soluble sugars. However, such a strong spectral sensitivity of Fe and Zn is less commonly emphasized in lettuce lighting studies, which tend to focus more on biomass, pigments, phenolics, nitrate, and antioxidant traits [53].

### 3.2. Lighting Photoperiod Effects

Several studies in CEA have already shown that improving the edible biomass of several indoor crops is possible by lowering light intensity over a longer photoperiod (compared to a higher-intensity light for a shorter duration) [54,55,56]. According to principal component analysis, the impact of different photoperiod and lighting intensity combinations at the same daily light integral was differentiated in *Chrysanthemum coronarium* greens (Figure 5b). The continuous 24 h light effect on chrysanthemum was separated from 16 and 12 h photoperiod impact due to variation in macronutrient, Fe, chlorophyll, organic acid, α tocopherol, and soluble sugar contents (Table S2). The effects of the 12 and 16 h photoperiods are distributed along the F2 axis and correspond to growth parameters, total phenolic compounds, and β-carotene. Because DLI was kept constant in the photoperiod experiment, the observed responses primarily reflect the effect of temporal light distribution. Under these conditions, the 12 h treatment produced the highest (53–35% higher than the 16 h treatment) leaf area, biomass, and LUE, indicating that shungiku used the same daily photon dose more efficiently when supplied over a shorter period at higher PPFD. This contrasts with several lettuce studies. Longer photoperiods at equal DLI increased daily electron transport through PSII in lettuce [57], and longer photoperiods with lower PPFD increased biomass in lettuce and mizuna [58]. Likewise, lettuce growth at a given DLI depended on the specific PPFD × photoperiod combination rather than on DLI alone [26,56]. This does not contradict lettuce-based studies so much as refine them: the biological effects of photoperiod at fixed DLI are crop-dependent, and shungiku appears to benefit more from a shorter, more intense photoperiod than from prolonged illumination. Photoperiod also altered measured biochemical traits independently of biomass. Both 12 h and 24 h increased protein and phenolics (~50% higher) relative to 16 h, while 24 h resulted in 50% higher α-tocopherol, and the 12 h photoperiod in 2.3- and 1.3-fold higher Fe and Zn. Consistent with the literature, these shifts indicate that the photoperiod/PPFD relationship in shungiku acts not merely as a means of delivering total DLI, but also as a biologically meaningful variable affecting carbon gain and biomass formation [20,56]. At the same time, the contrasting behavior of sugars, proteins, phenolics, and micronutrients indicates that “quality” should not be interpreted as a single unified trait, but rather as a set of partially independent responses to light regime.

### 3.3. Lighting Intensity Effects

Within the present set of separate experiments, the experiment on lighting intensity and *Chrysanthemum coronarium* produced a broad, integrated response across many measured traits, as also shown by PCA. The impact of lighting intensity (Figure 5c) is mainly attributed to the F1 component. It clearly distinguished the impact of lower light intensities (150 and 200 µmol m^−2^ s^−1^) from that of higher light intensities (250–300 µmol m^−2^ s^−1^), and this separation is reflected in the variation in most of the investigated variables (Table S2). Increasing PPFD from 150 to 300 µmol m^−2^ s^−1^ leaf area increased from 177 to 280 cm^2^, fresh weight from 11 to 17 g; dry weight from 0.6 to 1.1 g, soluble sugars were elevated about 40%, antioxidant activity by ~60%, as well as increased organic acid contents, and several micronutrients. These trends align with the findings and experiments on lettuce [59], confirming that higher light intensity positively impacts plant photosynthetic activity and biomass formation [60]. However, the addition of the LUE index clarifies that the yield-maximizing lighting intensity differs from the maximal efficiency. In the present study, 300 µmol m^−2^ s^−1^ produced the highest biomass, but the highest LUE occurred at 200 µmol m^−2^ s^−1^. Thus, increasing PPFD beyond a moderate level still improved yield, but not the efficiency of dry matter production per unit of supplied light. It is highly relevant for CEA, where electric lighting remains a major operational cost [61]. The financial viability of indoor agriculture depends on whole-system optimization, including efficient resource use and matching the right crop with specifically tailored, controllable environmental parameters [62]. Accordingly, the present LUE results should be interpreted as a biological efficiency indicator rather than a direct economic metric, because no explicit energy consumption or cost analysis was performed in this study. Nevertheless, adjusting LED spectral quality and intensity is essential for both biological and economic optimization of vertical farming systems [63]. However, the nutritional implications of leafy vegetables are equally important, as they define the value of CEA production [64]. *Chrysanthemum coronarium* (syn. *Glebionis coronaria*) is recognized as a rich source of proteins, antioxidant-active compounds, and minerals [35], but the present results show that this nutritional potential is strongly conditioned by light intensity. Low-intensity reduced Fe (by ~20% compared to 300 µmol m^−2^ s^−1^), Zn (by 64% compared to 300 µmol m^−2^ s^−1^), and Mn relative to the more productive treatments, suggesting that suboptimal lighting compromises both yield and micronutrient density. Research data also show that higher light intensity facilitates increased mineral absorption [65,66]; however, other studies indicate that higher PPFD might result in reduced Ca content [53], thereby increasing the risk of tipburn and underscoring the importance of case- and crop-specific selection of the optimal lighting intensity.

### 3.4. Comparison of the Impacts of Lighting Parameters

Taken together, the results confirm that spectrum, photoperiod, and intensity are not equal lighting settings, but distinct regulatory dimensions with different functional outcomes [43]. Because these three dimensions were examined in separate experiments, the present study does not provide a direct factorial comparison among them; rather, it offers a comparative and exploratory assessment of how each lighting dimension affected *Chrysanthemum coronarium* within its own experimental framework. Spectrum clearly altered the balance between growth and biochemical composition; photoperiod determined how efficiently a fixed DLI was translated into biomass and changed pigment and nutrient allocation; and intensity was associated with broad changes across productivity, metabolism, antioxidant response, and micronutrient accumulation. As discussed previously, it is partly in line with lettuce photoresponse to different lighting parameters: mixed spectra outperformed monochromatic light, higher light intensity and DLI promoted biomass, and no single light regime optimized growth and metabolic traits equally. Still, the present results show that lettuce-based lighting recommendations cannot be transferred directly to rarely cultivated leafy greens without species-specific validation. In addition, Fe and Zn were especially responsive to spectrum, photoperiod, and intensity in *Chrysanthemum coronarium*, suggesting that light regulation of mineral nutrient contents in leaves may represent an especially relevant response dimension in this crop.

From an applied perspective, the present results suggest that all three investigated lighting dimensions contributed differently to plant performance. Within the present experimental conditions, the spectrum appeared particularly relevant for balancing growth and biochemical traits; photoperiod modified the efficiency with which a fixed daily photon dose was utilized, and intensity produced the clearest biomass-associated responses, including marked shifts in several metabolic and mineral traits. Moreover, while artificial lighting is one of the main electricity cost drivers in CEA systems, regimes with higher apparent LUE (12 h photoperiod, moderate lighting intensity) may be biologically advantageous and potentially operationally relevant, even when they do not produce the highest absolute biomass. This confirms that shungiku greens, like lettuce and other leafy vegetables, require species-specific and trait-oriented lighting recipes rather than universal lighting conditions.

Future research should build on these directional findings by moving from separate lighting factor experiments toward integrated, multi-factor light design for *Chrysanthemum coronarium* and other underutilized leafy greens in CEA. In particular, the effects of PPFD and DLI should be experimentally disentangled, and the strong light sensitivity of Fe and Zn accumulation warrants mechanistic examination. The obtained results also suggest that a broader range of spectral compositions should also be evaluated, including wider spectra, white and far-red supplementation, and different red:blue ratios. Further work should also resolve the compound-specific basis of antioxidant responses, including the relative roles of individual phenolics, pigments, tocopherols, and other antioxidant-active metabolites, since total phenolic content did not consistently predict antioxidant activity. In addition, the contrasting responses of soluble sugars and proteins suggest that future studies should also examine carbon–nitrogen balance and its regulation under different light regimes. From an applied perspective, shungiku appears to be a promising crop for CEA diversification, but its commercial potential will depend on the development of lighting recipes based on plant photoresponse.

## 4. Materials and Methods

The experiments examined the response of chrysanthemum greens to different lighting parameters: spectrum, photoperiod, and intensity. The experiments were conducted in a walk-in, controlled-environment chamber. Basic growing conditions were maintained at 21/17 ± 2 °C day/night temperatures and 60–65% relative air humidity. Hydroponic nutrient solution was prepared from commercial concentrates (Plagron, Ospel, The Netherlands): Hydro a (NPK 3-0-1, Ca 4.2%, MgO 0.4%) and Hydro b (NPK 1-3-6, MgO 1.4%) were diluted with deionized water in a 1:1:400 ratio. pH was maintained at 5.5–6.0 and adjusted using H_2_SO_4_ or NaOH solutions.

Chrysanthemum (*Chrysanthemum coronarium*; *Asteraceae*; synonyms *Glebionis coronaria*, salad shungiku) (CN Seeds, Pymoor, UK) seeds were germinated in water-soaked rockwool cubes (20 × 20 mm, Grodan, Roermond, The Netherlands), one seed per rockwool cube. 10 days after germination, the seedlings were transplanted into 40 L deep-water culture (DWC) hydroponic tanks (12 plants per tank; at a density of 42 plants m^−2^).

Experimental design. Lighting treatments were explored in 3 parallel experiments. Each treatment consisted of three independent DWC tanks with individual light-emitting diode (LED) lamps and thus were treated as 3 biological replicates. For biometric traits, three plants per tank were measured as subsamples within biological replication. For biochemical traits, plant material from each tank was pooled into a single composite sample, yielding three biological replicates per treatment. Further, 3 extraction and 3 analytical repetitions were averaged before statistical analysis; therefore, ANOVA, correlation analysis, and PCA were based exclusively on biological replicate means.

Light spectra treatments consisted of (i) monochromatic red (R, peak wavelength 660 nm, full width at half maximum FWHM = 18 nm (LZ4-40R208 LEDs (Osram, Premstatten, Austria)), (ii) monochromatic blue (B, peak wavelength 447 nm, FWHM = 20 nm (LXML-PR02-A900 LEDs, Lumileds, Eindhoven, The Netherlands)), and (iii) combined RB (20% of B 447 nm and 80% of R 660 nm) lighting treatments (HLRD lighting units; Hortiled, Kaunas, Lithuania) at 16 h photoperiod and 250 µmol m^−2^ s^−1^ photosynthetically active photon flux density (PPFD) (daily light integral (DLI) 14.4 mol m^−2^). Monochromatic red and blue spectra were selected as mechanistic reference treatments to distinguish dominant wavelength-specific responses, while the 80% red/20% blue combination was chosen as a literature-based red-dominant benchmark commonly reported [23,24] as favorable for leafy vegetable cultivation under artificial lighting.

The lighting photoperiod experiment consisted of the same RB spectra (20% of B 447 nm and 80% of R 660 nm) at 3 lighting photoperiods (12, 16, and 24 h) with PPFD adjusted to 333, 250, and 167 µmol m^−2^ s^−1^, respectively, to maintain an equal DLI of 14.4 mol m^−2^ in all treatments.

The lighting intensity experiment consisted of 150, 200, 250, and 300 µmol m^−2^ s^−1^ PPFDs at a 16 h photoperiod, with DLI of 8.6, 11.5, 14.4, and 17.3 mol m^−2,^ respectively. Lighting spectrum in this experiment consisted of 61% of red (peak wavelength 660 nm, FWHM = 20 nm; GH CSSRM 4.24 LEDs (Osram, Premstatten, Austria)), 20% of blue (peak wavelenght 445 nm, FWHM = 18 nm; GH CSSRM 2.14 LEDs (Osram, Premstatten, Austria)), 15% of white (4000–4500 K; GW CSSRM 3.HW LEDs (Osram, Premstatten, Austria)) and 4% of far-red (730 nm peak wavelength, FWHM = 30 nm; GF CSHPM 2.24 LEDs (Osram, Premstatten, Austria). TUAS GTR 2V 0021096109 C1 DL ST lighting units (Tungsram, Budapest, Hungary) were used for lighting.

Reference in all experiments is RB spectra and 250 µmol m^−2^ s^−1^ PPFD, 16 h photoperiod. The PPFD was measured and controlled at the top of the plant using a spectrometer (WaveGo, Wave Illumination, Oxford, Oxfordshire, UK).

Measurements. Analyses were performed 35 days after sowing. All measurements in each experimental replication were performed in 3 analytical replications. Plant material was lyophilized (FD−7, SIA Cryogenic and Vacuum Systems, Latvia) and ground with the ultra-centrifugal mill ZM 300 (Retsch GmbH, Haan, Germany). Dry plant material was used to determine biochemical properties. The fresh (FW) and dry (DW); after lyophilization) shoot weights were determined (using laboratory scales with an accuracy of 0.001 g). The leaf area was measured using an automatic leaf area meter (Delta-T Devices, Wallingford, UK). The plant height was measured using a ruler with an accuracy of 1 mm.

Light use efficiency (LUE, mg mol^−1^ m^−2^) [67] was calculated as dry mass production per square meter per total incident light:

LUE = (DW × N)/(DLI × T)(1)

where DW—dry plant weight, N—number of plants per square meter, DLI—daily light integral per square meter, T—duration of cultivation cycle, days.

Antioxidant activity and total phenolic content determination. Antioxidant activity was expressed as DPPH (2-diphenyl-1-picrylhydrazyl), ABTS (2,2′-azino-bis (3-ethylbenzothiazoline-6-sulphonic acid) diammonium salt) free radical scavenging activity and ferric reduction antioxidant power (FRAP), and the total contents of phenolic compounds (TPCs) were evaluated in dry plant material. Lyophilized plant material (0.01 g) was ground with 5 mL of 80% methanol, incubated for 24 h, and centrifuged (10 min, 4500 rpm; Z366, Hermle, Germany), and the supernatant was used for analysis.

A stable 126.8 µM DPPH (100% purity; Sigma-Aldrich, Burlington, MA, USA) solution was prepared in methanol. Analyses were conducted in 96-well plates by mixing 280 µL of DPPH solution with 20 µL of plant extract in each well; the absorbance was read on a microplate reader at 515 nm (Spectro-star Nano, BMG Labtech, Germany) at the 16th minute. Results were expressed as µmol of DPPH scavenged per 1 g of dry plant weight (µmol g^−1^ DW) [68].

The ABTS radical solution was prepared by mixing 50 mL of 2 mM ABTS with 200 µL 70 mM K2S2O8, allowing the mixture to stand in the dark at room temperature for 16 h before use and diluting it to obtain an initial absorbance of AU 0.700 at 734 nm (M501, Camspec, Crawley, UK). Next, 100 µL of the sample was mixed with 2 mL ABTS solution, and absorbance was monitored for 11 min. The results are presented as ABTS free radical scavenging activity, µmol ABTS g^−1^ of plant DW [69].

The FRAP method is based on Fe^3+^ ion reduction to Fe^2+^. A fresh working solution was prepared by mixing the following solutions in a 10:1:1 volumetric ratio: 10 mM TPTZ (2,4,6-tripyridyl-s-triazine) solution in 300 mM, pH 3.6 acetate buffer, 40 mM HCl, and 20 mM FeCl_3_ × 6H_2_O. Analyses were conducted in 96-well plates by mixing 280 µL of working solution with 20 µL of plant extract in each well and incubating it in the dark for 30 min; the absorbance was read on a microplate reader at 593 nm (Spectro-star Nano, BMG Labtech, Ortenberg, Germany). Solutions of Fe_2_(SO_4_)_3_ in the range from 0.005 to 0.5 mM (Sigma-Aldrich, Burlington, MA, USA) were used to determine the calibration curve. Results were expressed as µmol of Fe^2+^ reduced by g^−1^ of dry plant weight (µmol g^−1^ DW) [70].

Analyses of total phenolics contents (TPC) were conducted by mixing 20 µL of plant extract with 20 µL of 10% (*v*/*v*) Folin–Ciocalteu reagent and 160 µL of 1 M Na_2_CO_3_ solution and incubating it in the dark for 20 min. Absorbance was read on a microplate reader at 765 nm (Spectro-star Nano, BMG Labtech, Germany). The contents of total phenolic compounds were quantified as gallic acid equivalents according to the calibration curve. Results were expressed by mg of TPC per dry plant weight (mg g^−1^ DW) [71].

Total protein contents were determined spectrophotometrically using the Bradford reagent (Sigma-Aldrich, Louis, MO, USA). Bovine serum albumin (0.05–1.0 mg mL^−1^) was used for quantification (Sigma-Aldrich, St. Louis, MO, USA). 10 μL of sample were mixed with 190 μL of diluted Bradford reagent, and after the 10 min incubation period, absorption at 595 nm was measured using a Spectro-star Nano microplate reader (BMG Labtech, Ortenberg, Germany). Protein content was presented as mg per g of plant dry weight (DW).

Nitrate/nitrite content determination was based on the Griess reaction. From sample preparation, dry plant material (0.05 g) was subjected to a hot water extraction at a ratio of 1:100 (*w*/*v*) on an orbital shaker for 30 min. Initial nitrite concentration and total nitrite after zinc reduction were determined by diazotizing with sulphanilamide and coupling with N-(1-naphthyl)-ethylenediamine dihydrochloride to form a highly colored azo dye with absorbance measured at 540 nm (Spectro-star Nano, BMG Labtech microplate reader, Germany). The nitrate content was calculated from the difference between the total nitrite content after reduction and the initial nitrite concentration. Nitrate and nitrite contents (mg kg^−1^) in dry plant weight (DW) were calculated by the external calibration method [72].

Sugar and organic acid analysis. For sample preparation, 0.1 g of freeze-dried plant material was weighed into 5 mL plastic microtubes (Th. Geyer GmbH & Co. KG, Renningen, Germany), mixed with 4 mL of warm (40–50 °C) deionized water, and then rotated for 2 h on the LABINCO LD79 digital test tube rotator (Labinco BV, Breda, The Netherlands). After extraction, the tubes were centrifuged at 4500 rpm in a Z366K centrifuge (HERMLE Labortechnik GmbH, Wehingen, Germany) for 15 min. Then, 1 mL of supernatant was filtered through a 0.22 µm nylon syringe filter and transferred to a chromatography vial for organic acid analysis. A total of 0.9 mL of supernatant was transferred into a 2 mL microtube, mixed with 0.9 mL of 0.01% ammonium acetate solution in acetonitrile, and stored in a refrigerator at 4 °C for 30 min. Samples were centrifuged at 14,000 rpm for 15 min in a MiniSpin centrifuge (Eppendorf AG, Hamburg, Germany), then filtered through 0.22 µm nylon syringe filters and transferred to 1.5 mL chromatography vials for sugar analysis. HPLC analyses of organic acids were performed on NEXERA HPLC (Shimadzu, Japan) with LCMS 2020 mass detector using YMC-Triart C18 column (150 × 3 mm, 3 µm) (YMC Europe GmbH, Dinslaken, Germany) operating at 40 °C, separations were performed using 0.2% of formic acid in water at a flow rate of 0.4 mL min^−1^. At the end of each chromatogram, the column was washed with 50% of acetonitrile for 5 min. HPLC analyses of sugars were performed on NEXERA HPLC (Shimadzu, Japan) with ELSD-LTII detector, using Shodex HILICpak VG-50 4D column (150 × 4.6 mm, 5 µm) (Shova Denko Europe GmbH, Munich, Germany), the separation was performed with water (A) and acetonitrile (B) at +35 °C and the flow rate of 0.8 mL min^−1^ was used. Gradient was formed as follows: initially 77% of solvent B was used, then in 11.5 min concentration of B was decreased to 71%, in the following 1.5 min gradient was returned to initial conditions and hold for 2 min. Sample injection volume of 10 µL was used [73].

The concentration of β carotene and chlorophylls [74] were evaluated by HPLC Extraction was performed using 80% aqueous acetone (0.05 g lyophilized material homogenized and diluted with 5 mL acetone solution; extraction was carried out for 24 h at 4 °C), then centrifuged (5 min, 4000× *g*) and filtrated through a 0.22 µm nylon membrane syringe filter (BGB Analytik, Boeckten, Switzerland). An HPLC 10A system (Shimadzu, Kyoto, Japan) with a diode array (SPD-M 10A VP) detector was used for analysis. Compounds were separated on a Chromegabond C30 (150 × 2.1, 3 µM) column (ES Industries, New Jersey, USA). Peaks were detected at 440 nm. The mobile phase consisted of A (80% methanol, 20% water) and B (100% ethyl acetate). Gradient: 0 min; 20% B, 2.5 min; 22.5% B, 20–22.5 min; 50% B, 24–26 min; 80% B, 31–34 min; 100% B, 42–47 min; and 20% B, flow rate 0.2 mL min^−1^. External calibration method was used for β carotene, chlorophyll a and b quantification (mg g^−1^ in DW).

Contents of α tocopherol were evaluated using the high-performance liquid chromatography (HPLC) method. Extracts were prepared by mixing about 0.05 g of dry lyophilized and homogenized plant material with 5 mL of hexane. An HPLC 10A system, equipped with an RF-10A fluorescence detector (Shimadzu, Japan) and EC NUCLEODUR 100-5 (150 × 4.6 mm, 5 µm) HPLC column (Macherey-Nagel GmbH & Co., Düren, Germany) was used for analysis. The mobile phase was 0.5% isopropanol (Merck, Germany) in n-hexane (isocratic elution); the flow rate was 1 mL min^−1^. Compounds was detected using an excitation wavelength of 295 nm and an emission wavelength of 330 nm. The identification of tocopherols was carried out by comparing the retention times of the peaks with the standard materials. The results are presented as mg g^−1^ in plant DW.

The contents of macro (P, K, Ca, Mg)- and microelements (Fe, Mn, Na, Zn) were determined using the microwave-assisted digestion technique [75] (Multiwave GO, Anton Paar, Graz, Austria), combined with inductively coupled plasma optical emission spectrometry [76] (Spectro Genesis, Kleve, Germany). Complete mineralization of 0.3 g dry plant material was achieved with 8 mL 65% HNO_3_. The digestion program was as follows: (1) 170 °C reached within 3 min, digested for 10 min; (2) 180 °C reached within 10 min, digested for 10 min. Mineralized samples were diluted to 50 mL with deionized water. The calibration curves for all the studied elements ranged from 0.01 to 400 mg L^−1^.

Statistical analysis. The results are presented as the average of 3 biological replicates for biochemical analysis and 3 subsamples per biological replicate for biometric analysis, for a total of *n* = 9 per treatment. A one-way ANOVA, using Tukey’s HSD test, was employed to determine differences between light treatments at the confidence level of *p* ≤ 0.05. For result modeling, a correlation analysis and a multivariate principal component analysis (PCA) were performed. Data were evaluated using Microsoft^®^ Excel^®^ for Microsoft 365 MSO (Version 2309 Build 16.0.16827.20166) 64–bit and compatible XLStat version 2022.1.2.1279 (64–bit) (Addinsoft, Paris, France) software packages.

## 5. Conclusions

We concluded that *Chrysanthemum coronarium* exhibits distinct and non-redundant photoresponses to lighting spectrum, photoperiod, and intensity under controlled-environment cultivation, indicating that these parameters regulate plant performance through different physiological dimensions rather than as interchangeable components of a single light regime. The observed dissociation among biomass formation, soluble sugars, antioxidant properties, mineral element accumulation, and light use efficiency further shows that plant response to artificial lighting is integrative but not uniform, and that no single lighting condition can be assumed to optimize all functional traits simultaneously. In this respect, *C. coronarium* should not be interpreted as a physiological equivalent of lettuce, despite their taxonomic relatedness and shared relevance for CEA, but rather as a crop with its own light response profile. Thus, the present study provides a physiological basis for species-specific light management in this underutilized leafy vegetable and supports a broader shift in controlled-environment agriculture from transferring standard lighting recipes toward designing crop-oriented production strategies.

## Figures and Tables

**Table 1:** Biometric indices in *Chrysanthemum coronarium* in response to different lighting parameters ($\bar{x},\;n=9\;\mathrm{subsamples}\;\mathrm{within}\;3\;\mathrm{biological}\;\mathrm{replications}$). Different letters indicate statistically significant differences between means of different lighting parameters in each experiment according to one-way ANOVA, Tukey’s test, when *p* ≤ 0.05.

	Height cm	Leaf Area cm^2^	Fresh Weight g	Dry Weight g	LUE mg mol^−1^ m^−2^
Lighting spectra					
R	12.5 ± 0.9 ^a^	72 ± 6 ^b^	5.2 ± 0.4 ^a^	0.30 ± 0.02 ^b^	29 ± 1 ^b^
B	14.5 ± 1.5 ^a^	111 ± 32 ^ab^	6.6 ± 2.0 ^a^	0.42 ± 0.11 ^ab^	41 ± 11 ^ab^
RB	13.5 ± 0.5 ^a^	143 ± 1 ^a^	8.0 ± 0.7 ^a^	0.51 ± 0.02 ^a^	50 ± 2 ^a^
Lighting photoperiod, h					
12	14.7 ± 0.3 ^a^	219 ± 40 ^a^	11.7 ± 0.1 ^a^	0.69 ± 0.08 ^a^	67 ± 7 ^a^
16	13.5 ± 0.5 ^ab^	143 ± 1 ^b^	8.0 ± 0.7 ^b^	0.51 ± 0.02 ^b^	50 ± 2 ^b^
24	12.2 ± 1.3 ^b^	140 ± 14 ^b^	8.9 ± 0.7 ^b^	0.51 ± 0.03 ^b^	50 ± 3 ^b^
Lighting intensity, µmol m^−2^ s^−1^					
150	14.2 ± 0.6 ^a^	177 ± 10 ^c^	10.9 ± 0.8 ^b^	0.57 ± 0.07 ^b^	80 ± 9 ^a^
200	13.7 ± 1.3 ^a^	200 ± 5 ^bc^	14.5 ± 1.5 ^ab^	0.81 ± 0.06 ^ab^	85 ± 6 ^a^
250	14.7 ± 0.9 ^a^	223 ± 5 ^b^	13.8 ± 0.9 ^ab^	0.80 ± 0.09 ^ab^	67 ± 8 ^b^
300	14.9 ± 0.9 ^a^	280 ± 22 ^a^	16.9 ± 2.9 ^a^	1.08 ± 0.23 ^a^	75 ± 16 ^ab^

**Table 2:** The lighting parameters impact on pigments content in *Chrysanthemum coronarium* ($\bar{x},\;n=3\;\mathrm{biological}\;\mathrm{replications})$. Different lowercase letters indicate statistically significant differences between means of different lighting parameters according to one-way ANOVA, Tukey’s test, when *p* ≤ 0.05.

	α Tocopherol µg g^−1^	Violaxanthin µg g^−1^	Lutein µg g^−1^	β Carotene µg g^−1^	Chlorophyll a mg g^−1^	Chlorophyll b mg g^−1^
Lighting spectra						
R	56.9 ± 9.5 ^a^	90.0 ± 7.4 ^a^	417 ± 6.5 ^a^	76.2 ± 1.4 ^a^	2.19 ± 0.03 ^a^	1.77 ± 0.01 ^a^
B	40.9 ± 7.5 ^a^	76.9 ± 10.2 ^ab^	359 ± 23 ^b^	81.2 ± 4.6 ^a^	2.25 ± 0.04 ^a^	1.68 ± 0.02 ^b^
RB	55.2 ± 3.4 ^a^	62.1 ± 8.8 ^b^	397 ± 18.3 ^ab^	65.8 ± 0.9 ^b^	2.26 ± 0.04 ^a^	1.72 ± 0.04 ^ab^
Lighting photoperiod, h						
12	69.1 ± 4.8 ^a^	73.4 ± 12.6 ^a^	348 ± 4.9 ^c^	51.1 ± 2.3 ^b^	1.98 ± 0.03 ^c^	1.64 ± 0.07 ^b^
16	55.2 ± 3.4 ^b^	62.1 ± 8.8 ^a^	397 ± 18.3 ^b^	65.8 ± 0.9 ^a^	2.26 ± 0.038 ^b^	1.72 ± 0.04 ^ab^
24	42.8 ± 3.7 ^c^	64.2 ± 7.7 ^a^	441 ± 16.3 ^a^	53.8 ± 7.6 ^b^	2.51 ± 0.095 ^a^	1.85 ± 0.06 ^a^
Lighting intensity, µmol m^−2^ s^−1^						
150	90.1 ± 3.5 ^a^	38.2 ± 2.4 ^a^	362 ± 17.4 ^a^	65.4 ± 7.2 ^a^	2.12 ± 0.09 ^a^	1.75 ± 0.04 ^a^
200	81.4 ± 13.3 ^a^	38.0 ± 4.2 ^a^	378 ± 16.8 ^a^	79.4 ± 10.8 ^a^	1.73 ± 0.04 ^c^	1.57 ± 0.04 ^b^
250	67.8 ± 14.5 ^a^	33.8 ± 1.2 ^a^	389 ± 18.8 ^a^	82.6 ± 1.8 ^a^	1.96 ± 0.08 ^ab^	1.64 ± 0.04 ^ab^
300	74.0 ± 19.9 ^a^	36.6 ± 8.3 ^a^	353 ± 16.1 ^a^	77.7 ± 2.1 ^a^	1.79 ± 0.05 ^bc^	1.42 ± 0.06 ^c^

**Table 3:** The effect of lighting parameters impact on macro- microelement concentrations in *Chrysanthemum coronarium* ($\bar{x},\;n=3\;\mathrm{biological}\;\mathrm{replications})$. Different lowercase letters indicate statistically significant differences between means of different lighting parameters according to one-way ANOVA, Tukey’s test, when *p* ≤ 0.05.

	Macroelements				Microelements			
	P mg g^−1^	K mg g^−1^	Ca mg g^−1^	Mg mg g^−1^	Fe µg g^−1^	Mn µg g^−1^	Na µg g^−1^	Zn µg g^−1^
Lighting spectra								
R	0.78 ± 0.02 ^a^	58.1 ± 1.0 ^a^	7.08 ± 0.22 ^b^	2.03 ± 0.09 ^a^	3.89 ± 0.79 ^b^	280 ± 14 ^a^	1172 ± 65 ^a^	13.7 ± 1.5 ^b^
B	0.78 ± 0.07 ^a^	61.2 ± 3.3 ^a^	7.68 ± 0.60 ^ab^	1.96 ± 0.15 ^a^	4.83 ± 2.02 ^b^	207 ± 18 ^b^	1134 ± 141 ^a^	16.6 ± 3.2 ^b^
RB	0.84 ± 0.04 ^a^	58.2 ± 1.7 ^a^	8.12 ± 0.33 ^a^	1.86 ± 0.09 ^a^	13.39 ± 4.91 ^a^	250 ± 11 ^a^	1061 ± 47 ^a^	27.4 ± 2.3 ^a^
Lighting photoperiod, h								
12	0.94 ± 0.06 ^ab^	63.0 ± 2.6 ^ab^	8.65 ± 0.56 ^ab^	1.79 ± 0.14 ^a^	11.94 ± 4.35 ^b^	338 ± 23 ^a^	1317 ± 134 ^a^	30.7 ± 1.8 ^ab^
16	0.84 ± 0.04 ^b^	58.2 ± 1.7 ^b^	8.12 ± 0.33 ^b^	1.86 ± 0.09 ^a^	13.39 ± 4.91 ^b^	250 ± 11 ^b^	1061 ± 47 ^a^	274 ± 2.3 ^b^
24	1.06 ± 0.06 ^a^	67.4 ± 2.4 ^a^	9.35 ± 0.45 ^a^	1.97 ± 0.09 ^a^	31.39 ± 3.10 ^a^	314 ± 15 ^a^	1187 ± 114 ^a^	36.8 ± 3.3 ^a^
Lighting intensity, µmol m^−2^ s^−1^								
150	0.85 ± 0.01 ^b^	47.3 ± 0.4 ^a^	10.28 ± 0.04 ^b^	1.95 ± 0.03 ^ab^	29.24 ± 0.11 ^c^	225 ± 1.2 ^b^	1575 ± 7 ^a^	32.8 ± 0.2 ^b^
200	0.96 ± 0.022 ^a^	46.6 ± 0.9 ^ab^	11.38 ± 0.17 ^a^	2.01 ± 0.03 ^a^	33.36 ± 2.21 ^bc^	269 ± 4.3 ^a^	1502 ± 13 ^a^	37.5 ± 0.8 ^b^
250	0.81 ± 0.01 ^b^	45.0 ± 0.8 ^ab^	9.84 ± 0.12 ^b^	1.76 ± 0.05 ^c^	39.86 ± 1.63 ^a^	276 ± 8.4 ^a^	1364 ± 32 ^b^	51.8 ± 2.4 ^a^
300	0.85 ± 0.07 ^b^	44.0 ± 1.7 ^b^	9.94 ± 0.69 ^b^	1.87 ± 0.07 ^bc^	36.79 ± 3.86 ^ab^	273 ± 12.6 ^a^	1202 ± 79 ^c^	53.7 ± 3.8 ^a^

## Data Availability

The raw data supporting the conclusions of this article will be made available by the authors upon request.

## Supplementary Materials

The following supporting information can be downloaded at: https://www.mdpi.com/article/10.3390/plants15091394/s1, Table S1: Correlation coefficients between investigated growth and metabolic parameters in *Chrysanthemum coronarium* greens under the exposure of different lighting parameters: A—lighting spectra, B—lighting photoperiod, C—lighting intensity; Table S2: The Principal component analysis factor loadings exploring lighting parameters impact on *Chrysanthemum coronarium*.

## Abbreviations

The following abbreviations are used in this manuscript:

B	Blue light
CEA	Controlled environment agriculture
DLI	Daily light integral
DW	Dry weight
FWHM	Full width at half maximum
LUE	Light use efficiency
PCA	Principal component analysis
PPFD	Photosynthetic photon flux density
R	Red light
RB	Combined red and blue light
TPC	Total phenolic compounds
